# Outstanding supercapacitive properties of Mn-doped TiO_2_ micro/nanostructure porous film prepared by anodization method

**DOI:** 10.1038/srep22634

**Published:** 2016-03-04

**Authors:** Xuewen Ning, Xixin Wang, Xiaofei Yu, Jianling Zhao, Mingli Wang, Haoran Li, Yang Yang

**Affiliations:** 1 School of Materials Science and Engineering, Hebei University of Technology, Tianjin 300130, China; 2 Nanoscience Technology Center, Department of Materials and Engineering, University of Central Florida, Orlando, Florida 32826, US

## Abstract

Mn-doped TiO_2_ micro/nanostructure porous film was prepared by anodizing a Ti-Mn alloy. The film annealed at 300 °C yields the highest areal capacitance of 1451.3 mF/cm^2^ at a current density of 3 mA/cm^2^ when used as a high-performance supercapacitor electrode. Areal capacitance retention is 63.7% when the current density increases from 3 to 20 mA/cm^2^, and the capacitance retention is 88.1% after 5,000 cycles. The superior areal capacitance of the porous film is derived from the brush-like metal substrate, which could greatly increase the contact area, improve the charge transport ability at the oxide layer/metal substrate interface, and thereby significantly enhance the electrochemical activities toward high performance energy storage. Additionally, the effects of manganese content and specific surface area of the porous film on the supercapacitive performance were also investigated in this work.

Supercapacitors, as electrical energy storage devices (generally called electrochemical capacitors) have attracted much attention owing to their longer cycle life, more rapid charging/discharging rates, and higher power, compared with conventional dielectric capacitors and batteries^1^,^2^,^3^,^4^. Such outstanding performance largely satisfies the higher requirements of practical applications in portable electronics, hybrid electric vehicles, and large industrial equipments, *etc*.^5^,^6^. The electrode material is a crucial factor affecting the performance of a supercapacitor^7^. Active electrode materials are mainly categorised into three types: carbon materials, conductive polymer materials, and transition metal oxides^8^,^9^. Transition metal oxides are of great interest nowadays due to their higher energy density and better reliability compared with others^10^.

Among the frequently-used transition metal oxides, manganese oxide is generally considered as one of the most promising candidates due to its high theoretical capacitance derived from reversible oxidation state changes during charging/discharging process^11^,^12^,^13^,^14^. However, it suffers from shortcomings of low specific surface area^15^, poor electrical conductivity^14^, and easy dissolution^16^,^17^, *etc.* To overcome these problems, efforts have been focused on further improvement of its capacitive properties by combining MnO_2_ with carbon^18^, TiO_2_^19^,^20^, Co_3_O_4_^21^, ZnO^22^,^23^, Fe_2_O_3_^24^, *etc.*

TiO_2_ is an important semiconductor material with good chemical stability, low cost, low toxicity, and environmentally friendly nature^25^,^26^. Whereas, when TiO_2_ is used as electrode material alone, its capacitance is relatively low, and compositing TiO_2_ with manganese oxides could considerably improve its capacitive performance. For example, the composite of TiO_2_ and MnO displays significantly higher capacity than that of TiO_2_^27^; the TiO_2_@MnO_2_ nanobelts and the Mn-doped TiO_2_ nanosheet-based spheres exhibits superior rate capability and outstanding cycling performance^28^,^29^.

Fabrication approaches of the composites affect their capacitance performance significantly. For instance, the hybrid electrode material, prepared through depositing MnO_2_ onto TiO_2_ nanotube arrays, obtains an areal capacitance of 213.2 mF/cm^2^,^7^,^30^,^31^,^32^,^33^. Whereas, such composite structure could not completely overcome the easily-dissolving disadvantage of MnO_2_. Mn-doped TiO_2_ nanotube arrays, prepared by anodizing of Ti-Mn alloys, could address that issue because manganese can be uniformly doped into the tubular structure, nonetheless, the Mn-doped nanotube arrays suffer from very small capacitance value^34^. In order to further improve the capacitance properties of composite oxide of manganese and titanium, in the present study, Mn-doped TiO_2_ micro/nanostructure porous film was prepared through anodizing a Ti-Mn alloy containing 10.0 wt% Mn under suitable conditions and superior capacitive properties with high areal capacitance of 1451.3 mF/cm^2^ and excellent cycling performance were achieved.

## Results and Discussion

**Figure 1:**
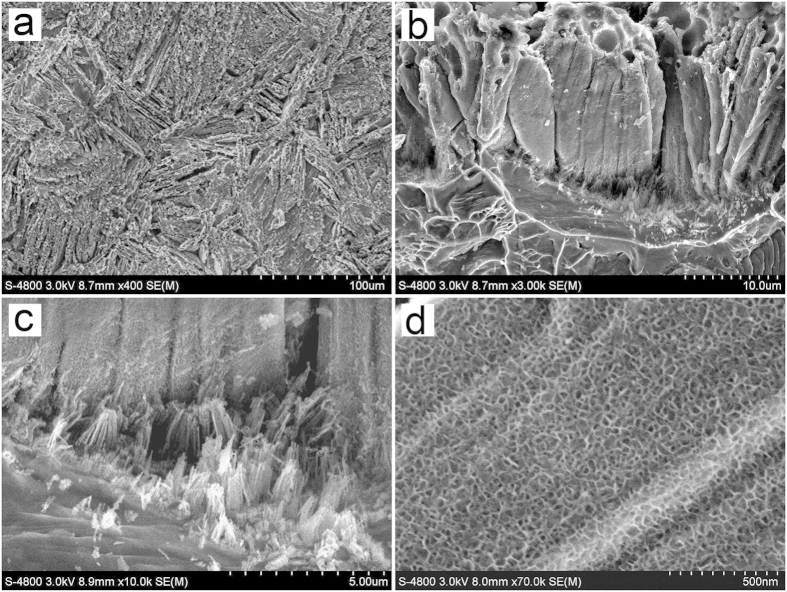
SEM images of the sample. (**a**) top view, (**b**) cross-sectional view, (**c**) interface between the oxide layer and alloy substrate, (**d**) nanoporous structure at the cylinder surface.

SEM images of the sample prepared through anodization of a Ti-Mn alloy in aqueous solution containing 0.25 wt% NH_4_F and 30 wt% ethylene glycol at 30 °C and 40 V for 3 h are shown in Fig. 1. The top view of the as-prepared sample displays a surface with criss-crossed gaps (Fig. 1a). The gaps extend from the surface to the alloy substrate, which divide the oxide into cylindrical structures at the micron-scale (Fig. 1b). Many fibrillar substances are left at the exposed surface of alloy substrate where cylindrical structures were peeled off (Fig. 1c), indicating that the oxide layer is closely connected to the substrate. Numerous nanopores were found on the surface of these cylindrical structures (Fig. 1d). In brief, under this condition, a type of micro/nanostructure porous film with micron-scale cylinders, whose surface is full of nanoporous structures, is obtained. This kind of micro/nanostructure porous film will be referred to as porous film hereinafter.

**Figure 2:**
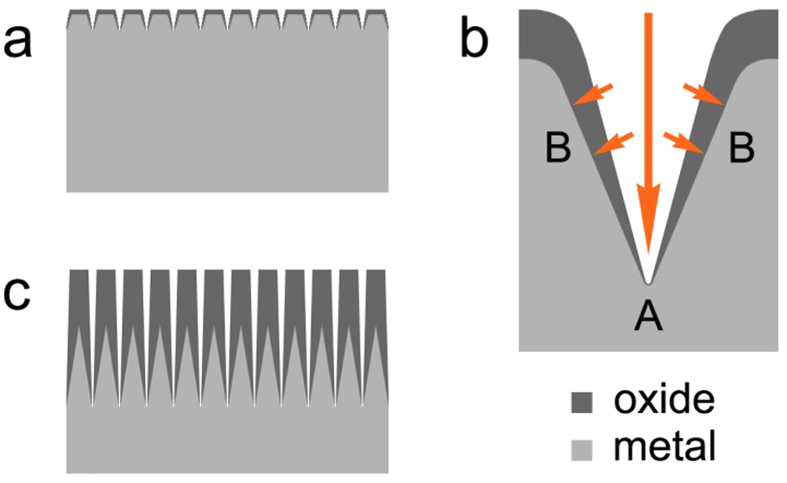
Schematic formation mechanism of Mn-doped TiO_2_ micro/nanostructure porous film (**a**) formation of tiny gaps, (**b**) state of the reaction in the tiny gaps, (**c**) structure of the oxide film.

Based on Fig. 1, formation mechanism of the micro/nanostructure porous film was proposed (Fig. 2). The microstructure of the alloy surface is irregular, thus, a non-uniform anodizing reaction and electrical breakdown occur at the early stage of anodization, which result in the formation of tiny gaps at the alloy surface (Fig. 2a). During the anodization process, the current density is as large as 100 mA/cm^2^. The larger curvature results in the higher charge density and faster reaction rate at the top of gaps (Fig. 2b, point A) than that at the side position (Fig. 2b, point B). Along with the increasing anodization time, the alloy surface becomes uneven due to the different reaction rates at the top and side of the gaps. Reactions at the top position lead to the formation of gaps, and reactions at the side position lead to the formation of nanoporous structures. Eventually, the porous film structure as shown in Fig. 2c is formed. On account of the faster reaction rate at point A than that at point B in Fig. 2b, there is unreacted metal (fibrillar substances as seen in Fig. 1c) inside the cylindrical structures after the reaction. The fibrillar substances are connected with the underneath metal and form the brush-like metal substrate (Fig. 2c).

**Figure 3:**
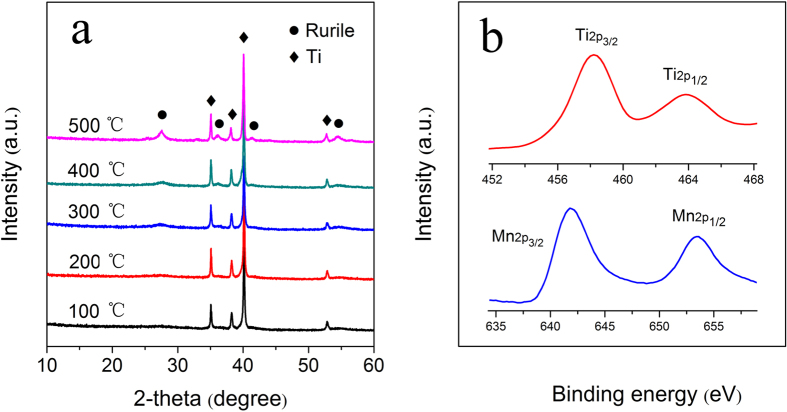
XRD patterns of Mn-doped TiO_2_ micro/nanostructure porous film annealed at different temperatures (**a**), XPS Ti2p and Mn2p spectra of the porous film (**b**).

To study the crystal structure of the oxide film, XRD analyses were conducted (Fig. 3a). When the sample was annealed at 100 °C and 200 °C, only those diffraction peaks of the titanium substrate (PDF card 44–1294) were detected, indicating that the oxides are amorphous. When the sample was annealed at 300 °C, 400 °C, and 500 °C, rutile TiO_2_ (PDF card 78–1510) is obtained. At 300 °C, rutile TiO_2_ was newly detected with weak characteristic diffraction peaks, indicating the formation of a small amount of TiO_2_ microlite in the oxide film. When annealed at 400 °C and 500 °C, the crystallinity of rutile TiO_2_ is improved. No characteristic peaks of manganese oxide can be seen in Fig. 3a, indicating that elemental Mn is scattered in the TiO_2_ matrix. To study the elemental valence of the oxides, XPS measurement results are given in Fig. 3b. The characteristic peaks of Ti2p_3/2_ and Ti2p_1/2_ are located at about 458.5 eV and 464.0 eV, respectively, confirming that Ti element is in Ti^4+^ state^2^,^35^. The peaks of Mn2p_3/2_ and Mn2p_1/2_ appears at about 641.6 eV and 653.3 eV, indicating that Mn element is in its +4 oxidation state (Fig. 2c)^10^,^36^.

**Figure 4:**
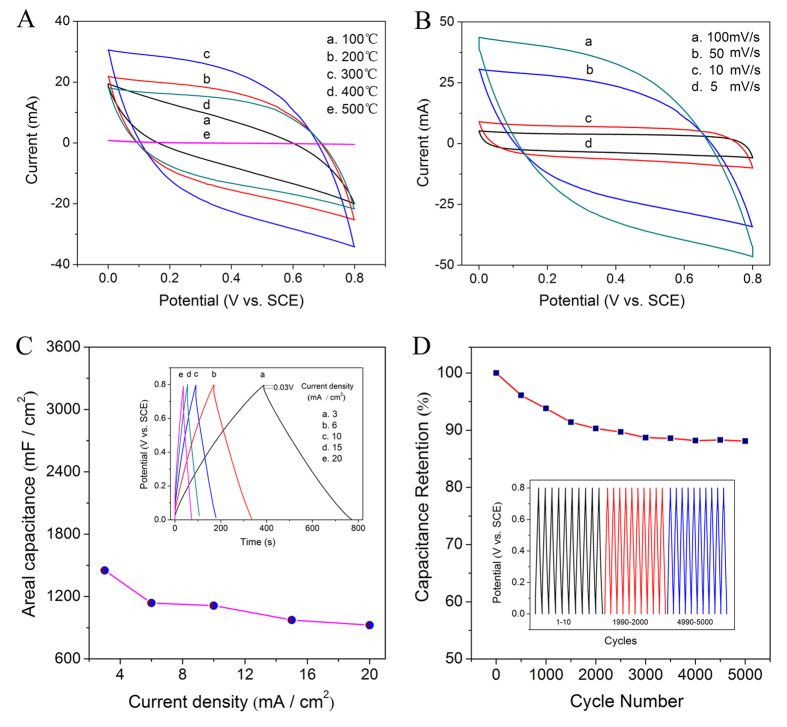
CV curves of the porous film at different annealing temperatures (scan rate: 50 mV/s) (**A**) and scan rates (annealing temperature: 300 °C) (**B**); Areal capacitance as a function of current density (the inset shows the GCD curves at different current densities) (**C**) and cycling performance at a current density of 10 mA/cm^2^ over 5,000 cycles (**D**).

The cyclic voltammetry (CV) curves of the porous film annealed under different temperatures ranging from 100 to 500 °C are shown in Fig. 4A. Along with the increasing annealing temperatures, the integrated area of the CV curves first increases and then decreases. The integrated area is maximised at 300 °C, and the area is minimised at 500 °C. The influence of annealing temperature is manifested in two ways: along with the increasing annealing temperature, on the one hand, improved atomic order degree and the formation of rutile TiO_2_ would lead to the enhanced conductivity and the increased utilisation rates of active materials, therefore, the capacitive value would increase accordingly^2^; on the other hand, mutual condensation reaction of hydroxyl groups would result in the reduced specific surface area^37^,^38^ and the decreased hydrophilicity, hence, the capacitive value would decrease accordingly. The two opposite effects of annealing temperatures on the capacitive properties result in that the highest capacitive value appears when the samples were annealed at 300 °C (Fig. 4A). Therefore, the porous film annealed at 300 °C is chosen for subsequent measurements.

Figure 4B shows the CV curves of the porous film annealed at 300 °C under different scan rates. According to Fig. 4B, the areal capacitance is 188.2, 169.5, 108.0, and 71.5 mF/cm^2^ at scan rates of 5, 10, 50, and 100 mV/s, respectively. The galvanostatic charge-discharge (GCD) measurements and corresponding areal capacitance are shown in Fig. 4C. At a current density of 3 mA/cm^2^, the highest areal capacitance of 1451.3 mF/cm^2^ was obtained. The retention of capacitance is 85.3%, 76.7%, 67.2%, and 63.7% at current densities of 6, 10, 15, and 20 mA/cm^2^ respectively. The charge curve is nearly symmetrical to the discharge curves, and the corresponding IR drop is 0.03 V when the current density is 3 mA/cm^2^ (inset, Fig. 4C). Figure 4D shows the continuous GCD curves of porous film at a current density of 10 mA/cm^2^ over 5,000 cycles. The capacitance retention is 90.3% after 2,000 cycles, and 88.1% after 5,000 cycles.

From Fig. 4C, Mn-doped TiO_2_ porous film obtains the highest areal capacitance of 1451.3 mF/cm^2^ at a current density of 3 mA/cm^2^. For comparison, the recently reported areal capacitance values obtained from the composites of MnO_2_ and TiO_2_ at the same current density are summarised in Table 1. Moreover, the value is also higher than those previously reported areal capacitances at lower current densities. For example, the MnO_2_-TiN nanotube^7^ achieves its highest areal capacitance of 213.2 mF/cm^2^ at a current density of 0.25 mA/cm^2^ and TiO_2_@MnO_2_ nanowall arrays^31^ achieves their highest specific capacitance of 15.5 mF/cm^2^ at a current density of 0.01 mA/cm^2^.

**Figure 5:**
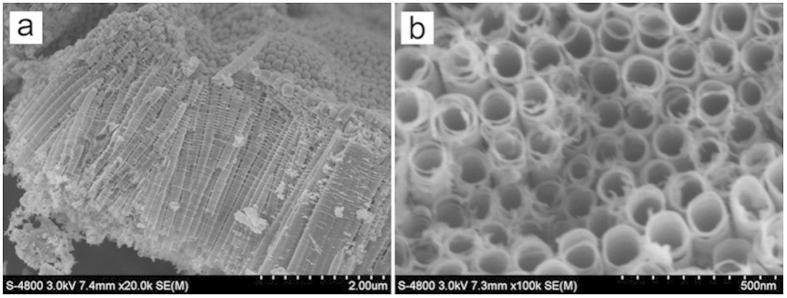
SEM images of Mn-doped TiO_2_ nanotube arrays (**a**) cross-sectional view; (**b**) top view.

**Figure 6:**
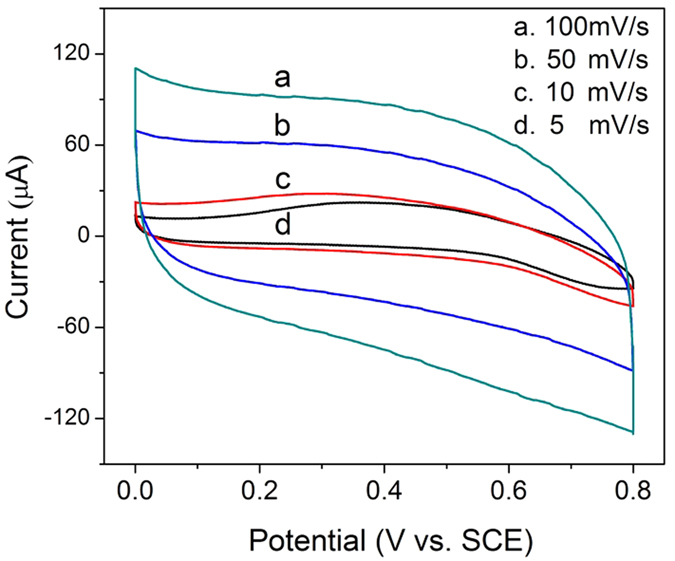
CV curves of Mn-doped TiO_2_ nanotube arrays at different scan rates (annealing temperature: 300 °C).

In order to compare the properties of this porous film with that of the frequently studied nanotube arrays, by changing the experimental conditions, Mn-doped TiO_2_ nanotube arrays were prepared through anodizing the same Ti-Mn alloy in ethylene glycol electrolyte containing 0.25 wt% NH_4_F and 5 wt% H_2_O at 30 °C and 40 V for 3 h. The SEM images are shown in Fig. 5. The nanotube arrays have an outer diameter of about 110 nm, a wall thickness of about 20 nm and a length of about 3.5 μm. The CV curves of Mn-doped TiO_2_ nanotube arrays tested under same conditions are shown in Fig. 6. The calculated areal capacitances are 0.63, 0.32, 0.24, and 0.21 mF/cm^2^ at scan rates of 5, 10, 50, and 100 mV/s, respectively. According to Figs 4B and 6, the areal capacitance of the porous film is about 300 times higher than that of the Mn-doped TiO_2_ nanotube arrays.

In order to study the reason why the porous film possesses such a high areal capacitance, manganese content and specific surface area of Mn-doped TiO_2_ porous film and Mn-doped TiO_2_ nanotube arrays were analyzed (Table 2). Results show that the specific surface area of the porous film and nanotube arrays is similar, while the manganese content differs a lot. Manganese content of porous film is about 2.25 times higher than that of nanotube arrays, which might be due to the different solubility of manganese oxide and titanium oxide in different electrolytes. The different manganese content would lead to different capacitance value, however, the fairly high capacitance value of porous film which is about 300 times of that of nanotube arrays could not be only ascribed to its nearly doubled manganese content.

**Figure 7:**
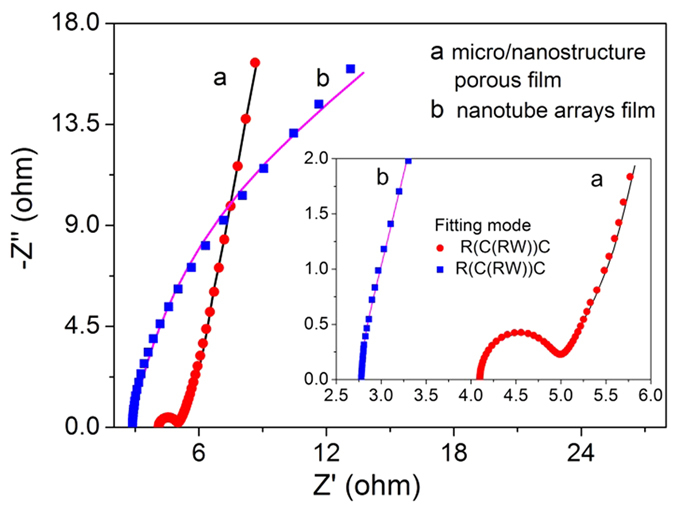
Impedance Nyquist plot of samples and the fitted results using a ZVIEW electrochemical software.

Electrochemical impedance spectroscopy (EIS) analysis is an effective method of explaining the capacitance performance of materials. Impedance Nyquist plots and equivalent circuits of porous film and nanotube arrays are shown in Fig. 7. The simulation data, according to the equivalent circuits, matches the experimental data very well. The semicircular arc in the EIS plot is observed for porous film at high-frequency and is followed by a sloping line at low-frequency. A sloping line is also observed for the nanotube arrays, but they lack the corresponding semicircular arc. The difference in EIS results indicates that the electrochemical processes in porous film are controlled by a combination of ion diffusion and electrochemical reaction, and the electrochemical processes in nanotube arrays is mainly controlled by ion diffusion. According to the simulation results, the charge transfer resistance and diffusion resistance admittance coefficient (*Y*_0_) of porous film are 0.23 Ω/cm^2^ and 0.17 S•sec^0.5^/cm^2^, respectively. Whereas, for nanotube array film, the diffusion resistance admittance coefficient (*Y*_0_) is 0.00060 S•sec^0.5^/cm^2^. The diffusion resistance admittance coefficient of porous film is about 283.3 times of that of nanotube arrays. Consequently, small diffusion resistance might be the main reason for the high areal capacitance of porous film.

The diffusion resistance is closely related with the morphology and structure of the hybrid electrode. According to Figs 1 and 2, the brush-like alloy substrate of the porous film would greatly improve the contact area and conductive ability between oxide layer and the metal substrate, and therefore, significantly reduce the charge transfer resistance, increase the utilisation rate of the active materials and consequently result in the outstanding areal capacitance value. For nanotube arrays film (Fig. 5), only the tube bottom is connected with metal substrate and the charge transfer resistance along with the nanotube wall is very large, resulting in the worse capacitance properties for the nanotube arrays in comparison with the porous film.

In summary, Mn-doped TiO_2_ micro/nanostructure porous film was successfully prepared by a simple anodization method. The annealing temperature exerts a significant influence on the capacitive performance of the porous film. The film annealed at 300 °C yields the highest areal capacitance of 1451.3 mF/cm^2^ at a current density of 3 mA/cm^2^. Areal capacitance retention is 63.7% when the current density increases from 3 to 20 mA/cm^2^, and the capacitance retention is 88.1% after 5,000 cycles. The capacitance performance of porous film is much better than that of the nanotube arrays film. The enhanced capacitance properties of the porous film might be mainly ascribed to specific preparation method and unique morphology structure. The brush-like alloy substrate of the porous film would greatly improve the contact area and conductive ability between oxide layer and the metal substrate, and therefore, significantly reduce the charge transfer resistance and increase the utilisation rate of the active materials. Additionally, manganese content and specific surface area of porous film also have a certain influence on its performance.

## Methods

Ti-Mn (Mn: 10.0 wt%) alloy prepared using a power metallurgical method was cut into 20 mm × 40 mm × 2 mm foils, and then polished with metallographic abrasive paper, and ultrasonically washed in twice-distilled water and ethanol before use. Anodization was performed using a program-controlled DC source (Dahua Coop., Beijing, China). The anodization set-up consisted of a two-electrode configuration with Ti-Mn alloy foil as the anodic electrode and platinum foil (30 mm × 50 mm × 0.1 mm) as the cathodic electrode. All anodization experiments were conducted in aqueous solution containing 0.25 wt% NH_4_F and 30 wt% ethylene glycol at 30 °C and 40 V for 3 h. After anodization, the samples were rinsed in deionised water, air-dried, and characterised.

**Figure 8:**
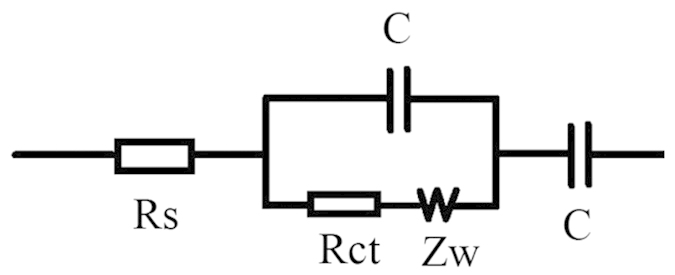
The corresponding equivalent circuit model.

Morphologies, structures, elemental valence state, manganese content, and specific surface area of the samples were investigated using a scanning electron microscope (SEM, Hitachi, S-4800), an X-ray diffractometer (XRD, Rigaku, D/MAX-2500), an X-ray photoelectron spectroscope (XPS, ULVAC-PHI, PHI-5000 VersaProbe), and an inductively coupled plasma optical emission spectrometer (ICP-OES, Leemon, Prodigy Xp), and specific surface area analyser (BET, Quantachrome Corportion, Autosorb Iq), respectively. The electrochemical properties of the samples were investigated by cyclic voltammetry (CV) and galvanostatic charge-discharge (GCD) testing using an electrochemical analyser (CH Instruments, Inc. USA, CHI660E). The electrochemical measurements were conducted in a standard three-electrode configuration comprising the sample as the working electrode, a platinum foil as the counter-electrode, a saturated calomel electrode (SCE) as the reference electrode, and 0.5 M Na_2_SO_4_ as the electrolyte. The analysis of the capacitive performance of each sample was based on the geometric area of the working electrode^2^,^14^. EIS measurements were tested in the range of frequencies from 0.01 Hz to 100 kHz at open potential and an alternating current (AC) voltage amplitude of 5 mV. Figure 8 shows the corresponding equivalent circuit model.

## Additional Information

**How to cite this article**: Ning, X. *et al*. Outstanding supercapacitive properties of Mn-doped TiO_2_ micro/nanostructure porous film prepared by anodization method. *Sci. Rep.* **6**, 22634; doi: 10.1038/srep22634 (2016).

## Figures and Tables

**Table 1:** Areal capacitance (mF/cm^2^) of the composites of MnO_2_ and TiO_2_ as a function of the current density (*I*, mA/cm^2^).

***I***	A*	B*	C*	**This work**
3	105	175	50	1451.3
6	80	150	40	1237.5
10	/	/	30	1112.5
15	/	/	25	975.0
20	/	/	24	925.0

^*^A: MnO_2_ spheres deposited on planar Ti^32^, B: MnO_2_ spheres deposited on TiO_2_ NTAs^32^, C: MnO_2_-TiN nanotube^7^.

**Table 2:** Manganese content, specific surface area (SSA) and capacitance of the samples.

**Samples**	**SSA (m**^**2**^**/g)**	**Mn/(Mn + Ti) (wt%)**	**C (mF/cm**^2^)
Mn-doped TiO_2_ nanotube arrays	62.89	5.44	0.63
Mn-doped TiO_2_ porous film	63.76	12.23	188.2

*The area capacitance value (C) was calculated from CV curves at a scan rate of 5 mV/s.
